# A Multicenter Observational Study for the Establishment of Novel Severity Criteria Including Endoscopic Evaluation for Intestinal Behçet's Disease

**DOI:** 10.1111/den.70041

**Published:** 2025-09-27

**Authors:** Toshiro Fukui, Makoto Naganuma, Yohei Kirino, Reiko Kunisaki, Yohei Mikami, Nobuhiro Ueno, Junji Umeno, Shigeki Bamba, Makoto Ooi, Shuhei Hosomi, Takayuki Matsumoto, Katsuyoshi Matsuoka, Chikako Watanabe, Masakazu Nagahori, Motoi Uchino, Kenji Watanabe, Fumihito Hirai, Minoru Matsuura, Yoshiya Tanaka, Mitsuhiro Takeno, Tadakazu Hisamatsu

**Affiliations:** ^1^ Division of Gastroenterology and Hepatology, Third Department of Internal Medicine Kansai Medical University Hirakata Japan; ^2^ Department of Stem Cell and Immune Regulation Yokohama City University Graduate School of Medicine Yokohama Japan; ^3^ Inflammatory Bowel Disease Center Yokohama City University Medical Center Yokohama Japan; ^4^ Division of Gastroenterology and Hepatology, Department of Internal Medicine Keio University School of Medicine Tokyo Japan; ^5^ Division of Gastroenterology, Department of Internal Medicine Asahikawa Medical University Asahikawa Japan; ^6^ Department of Medicine and Clinical Science, Graduate School of Medical Sciences Kyushu University Fukuoka Japan; ^7^ Department of Gastroenterology Shiga University of Medical Science Otsu Japan; ^8^ Department of Gastroenterology Kobe University International Clinical Cancer Research Center Kobe Japan; ^9^ Department of Gastroenterology Graduate School of Medicine, Osaka Metropolitan University Osaka Japan; ^10^ Division of Gastroenterology and Hepatology, Department of Internal Medicine, School of Medicine Iwate Medical University Morioka Japan; ^11^ Division of Gastroenterology and Hepatology, Department of Internal Medicine Toho University Sakura Medical Center Chiba Japan; ^12^ Department of Gastroenterology, Mita Hospital International University of Health and Welfare Tokyo Japan; ^13^ Health Science Research and Development Center, Institute of Science Tokyo Hospital Tokyo Japan; ^14^ Department of Gastroenterological Surgery Hyogo Medical University Nishinomiya Japan; ^15^ Department of Internal Medicine for Inflammatory Bowel Disease Toyama University Hospital Toyama Japan; ^16^ Department of Gastroenterology Faculty of Medicine, Fukuoka University Fukuoka Japan; ^17^ Department of Gastroenterology and Hepatology Kyorin University School of Medicine Mitaka Japan; ^18^ The First Department of Internal Medicine University of Occupational and Environmental Health Kitakyushu Japan; ^19^ Department of Allergy and Rheumatology Nippon Medical School Musashi Kosugi Hospital Kawasaki Japan

**Keywords:** anti‐TNF‐α treatment, disease activity, endoscopy, intestinal Behçet's disease

## Abstract

**Objective:**

This study aimed to establish a novel severity classification for intestinal Behçet's disease (BD) (SCIBD) and validate its criteria across multiple institutions.

**Methods:**

Five parameters, including abdominal pain, tenderness, intestinal bleeding, serum C‐reactive protein (CRP) level, and endoscopic findings, were identified to assess the severity of intestinal BD. Disease severity was categorized into remission and mild, moderate, or severe disease based on the criteria of each factor. This study also evaluated the correlation among the SCIBD scale, serum biomarkers, former disease activity for intestinal BD (DAIBD), and treatment decisions.

**Results:**

A total of 146 patients with intestinal BD and simple ulcers were retrospectively enrolled from 14 institutions between April and November 2022. As SCIBD severity increased, CRP and DAIBD levels significantly increased, whereas serum albumin levels decreased in the whole population. Similar correlations have been observed even in patients with intestinal BD. Antitumor necrosis factor‐alpha treatment was also significantly more common in severe cases (49.4%) than in moderate cases (20.8%; *p* = 0.001). However, the proportion of patients requiring corticosteroids was comparable between the moderate and severe disease groups (39.6% vs. 33.3%). In addition, no significant differences were observed in the frequency of corticosteroid treatment, anti‐TNF‐α treatment, or surgery among the four groups: quiescent, mild, moderate, and severe cases of DAIBD. SCIBD was changed after treatment with corticosteroids and TNF‐α according to improving clinical, biological, and endoscopic findings.

**Conclusions:**

The severity assessment of intestinal BD using our novel criteria correlated with appropriate treatment decisions, prognosis prediction, and treatment responses.

## Graphical abstract

The establishment of novel severity criteria for intestinal Behçet's disease.

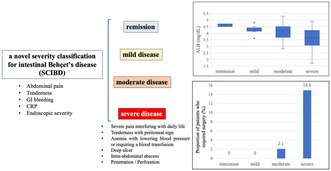

## 1 Introduction

Behçet's disease (BD) is an intractable disease of unknown etiology characterized primarily by recurrent oral aphthoid ulcers, ocular symptoms, skin lesions, and genital ulcers. Intestinal BD presents with characteristic circular or oval, well‐defined, and deep ulcers in the ileocecal region, accompanied by abdominal symptoms such as abdominal pain and bloody stools [1, 2, 3, 4]. Although cases that do not meet the criteria for complete or incomplete BD may exhibit similar ulcerative lesions in the ileocecal region, these lesions are classified as simple ulcers according to the current criteria, which reflect disease concepts unique to Japan. In clinical practice, intestinal BD and simple ulcers are considered to belong to the same disease entity and are often treated as pathophysiologically related diseases [5]. Although severity criteria for BD exist in Japan [3], there is currently no clear definition of severity specific to intestinal BD. Previous treatment guidelines for intestinal BD indicate that 5‐aminosalicylates are used for mild‐to‐moderate disease, whereas corticosteroids and antitumor necrosis factor‐alpha (anti‐TNF‐α) treatment are used for moderate‐to‐severe disease [4]. However, the severity of intestinal BD remains poorly defined, limiting our ability to select appropriate treatment strategies based on the disease severity. Therefore, the present study aimed to evaluate the validity of a severity classification system (Severity Criteria for Intestinal BD; SCIBD) based on expert consensus across multiple institutions.

## 2 Methods

### 2.1 Study Design

This multicenter retrospective cohort study aimed to validate the criteria for assessing intestinal BD severity. The severity criteria were developed by a committee led by Chief Investigator Makoto Naganuma from Kansai Medical University between April 2021 and November. After several meetings among the committee members, a final draft was drafted. After obtaining approval from the research members of the Health and Labor Sciences Research Grants for Research on Intractable Diseases and Research on BD from the Ministry of Health, Labor, and Welfare of Japan, the severity criteria were made public in March 2022.

To analyze the developed severity criteria, treatment decisions, and prognosis, patients' clinical information on intestinal BD and simple ulcers was retrospectively collected from 14 institutions between April and November 2022 using medical charts from each institution. Data were collected from patients who initially underwent colonoscopy and who were diagnosed with intestinal BD or simple ulcers. Intestinal BD was defined as the presence of characteristic oval, volcano‐shaped ulcerative lesions in the ileocecal region that met the Japanese diagnostic criteria for complete or incomplete BD [4]. Simple ulcers were defined as typical ulcerative lesions in the ileocecal region in patients who did not meet the diagnostic criteria for complete or incomplete BD [5, 6].

Clinical information was collected from the medical charts of patients with intestinal BD (both complete and incomplete) and simple ulcers.

All patients underwent lower gastrointestinal endoscopy both before and after therapeutic intervention. A colonoscopy was performed between January 2006 and June 2022. The following patients were excluded from enrollment: Those judged to be inappropriate by the researchers, those who declined to participate in this study, and those with atypical features but without ileocecal lesions.

### 2.2 Establishment of Severity Criteria for Intestinal BD

**FIGURE 1:**
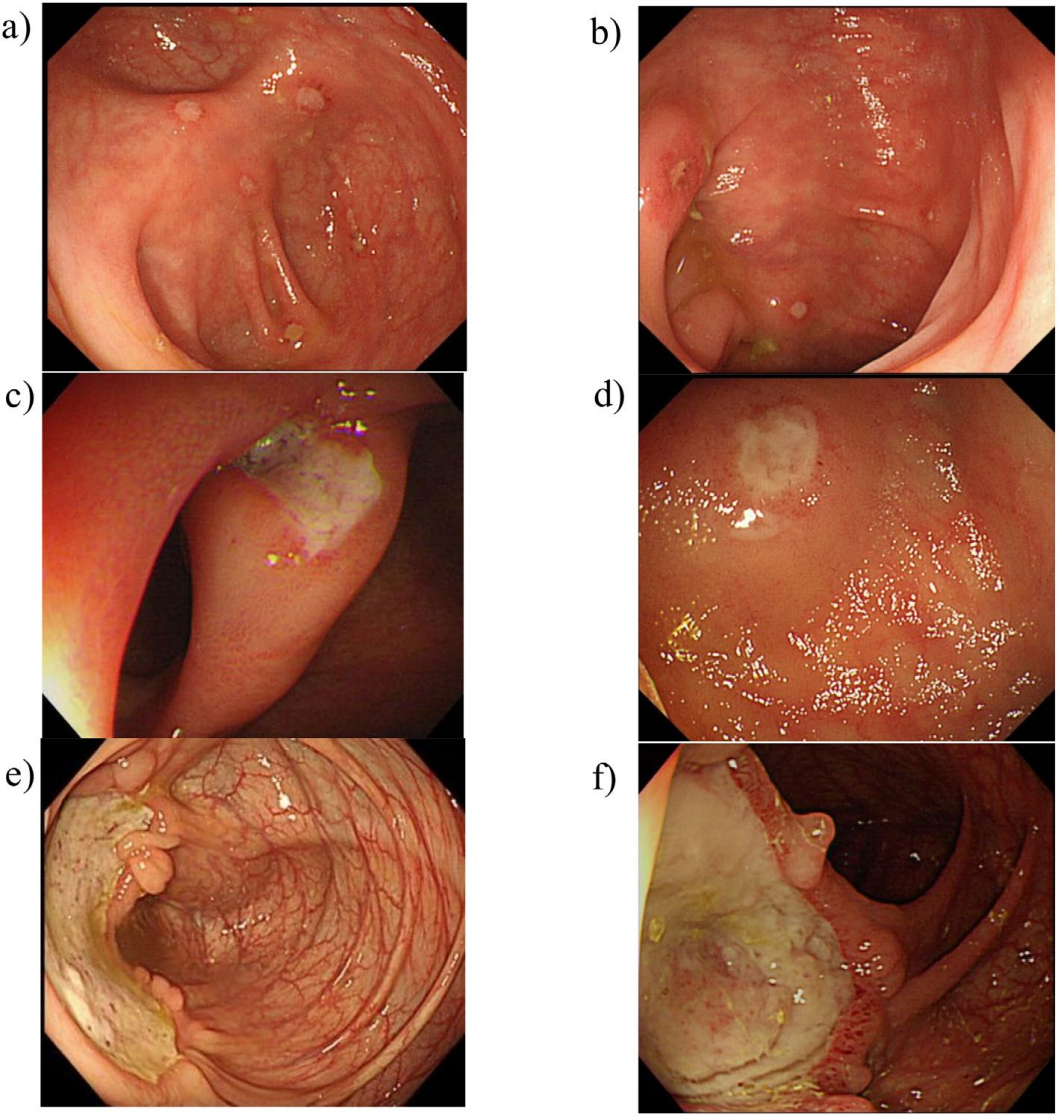
Typical endoscopic findings in patients with Grade 1 (a, b), Grade 2 (c, d), and Grade 3 (e, f).

The Severity Criteria for Intestinal BD (SCIBD) were established by committee members (Table 1). Abdominal pain and intestinal bleeding were selected as subjective symptoms, whereas abdominal tenderness, serum CRP levels, and endoscopic findings were chosen as objective indicators. Regarding endoscopic findings, aphthae and ulcers less than 1 cm in size were defined as Grade 1, whereas well‐demarcated, shallow ulcers ≥ 1 cm were defined as Grade 2 (Figure 1). For details on the creation process, refer to the supplemental information.

**TABLE 1:** Criteria for severity criteria for intestinal BD (SCIBD).

	Pain^a^	Tenderness^a^	Bleeding^a^	CRP (mg/dL)	Ulcer^b^
Grade 0	None	None	None	Below standard	None (including only scar lesions)
Grade 1	Mild (not interfering with daily life)			Greater than standard to less than 1.0	Aphthae and ulcers less than 1 cm
Grade 2	Moderate (sometimes interfering with daily life)	Without peritoneal signs	Overt	1.0 or higher	Well‐demarcated shallow ulcers of 1 cm or more (circular, quasi‐circular, irregular, geographic, etc.)
Grade 3	Severe (interfering with daily life)	With peritoneal signs	Massive (lowering blood pressure or requiring a blood transfusion)		Deep (‐mining) ulcer^c^
	Intra‐abdominal abscess Penetration/Perforation				

*Note:* Remission: Satisfying all 5 items of Grade 0. Mild: Satisfying at least one item of Grade 1, but not including Grade 2 or higher items. Moderate: Satisfying at least one item of Grade 2, but not including severe items. Severe: Satisfying at least one item of Grade 3.

^a^ Only those derived from gastrointestinal lesions in intestinal Behçet's disease.

^b^ In principle, judgment should be made including endoscopy, unless the severity is high and endoscopy is dangerous. If there are multiple ulcer lesions, evaluate the lesion with the highest grade (including lesions other than the ileocecal region).

^c^ Deep (‐mining) ulcer: Well‐demarcated deep ulcer with cliff‐cut margin.

### 2.3 Data Collection

Clinical information was collected at the time of the first colonoscopy. SCIBD and disease activity in intestinal BD (DAIBD) have also been assessed [7]. In DAIBD, the following were evaluated: general status, fever, extraintestinal findings, degree of abdominal pain within a week, presence of an abdominal mass, degree of tenderness, intestinal complications, and the number of times a patient passed watery stools within a week. In this study, there was no strict allowance on the interval between the date of the endoscopic procedure and the date of clinical information collection. However, each investigator was asked to collect clinical information closest to the date of the endoscopic procedure.

Treatment decisions, including the use of corticosteroids and anti‐TNF‐α treatment, were assessed approximately within a month after colonoscopy, along with the necessity for intestinal resections due to intestinal BD during the observation period.

### 2.4 Outcomes

The primary outcomes included the relationship between SCIBD and treatment decisions, as well as the rates of surgery. The secondary outcomes were the relationships between SCIBD and DAIBD at baseline and between SCIBD and serological severity at baseline. For the sensitivity analysis, we evaluated the validation of disease severity not only for intestinal BD but also for broadly defined intestinal BD, including suspected BD and simple ulcers (referred to the whole population in this study).

### 2.5 Statistical Analyses

Categorical variables were described using absolute numbers, and relative frequencies were expressed as percentages, whereas continuous variables were summarized as median and interquartile range (IQR). The median levels of blood biomarkers and DAIBD were compared among patients in each SCIBD using Kruskal–Wallis analysis. The proportions of patients treated with corticosteroids, anti‐TNF‐α treatment, and requiring surgery in severe disease were compared among patients with remission, mild disease, moderate, and severe disease using the chi‐squared or Fisher's exact test. All statistical analyses were performed using the International Business Machine (IBM) Statistic Package for the Social Sciences software (version 29.0; IBM Corp., Armonk, New York, United States), with statistical significance set at *p* < 0.05.

## 3 Results

### 3.1 Comparison of Blood Biomarkers With Disease Severity Before Treatment

We collected the clinical information related to the initial endoscopy procedure for each case. There were no cases in which ileocecal resection was performed in this study. Clinical characteristics and concomitant medications used in the study population are summarized in Table 2. Among the patients, 18 (12.3%) were diagnosed with simple ulcers, 26 (17.8%) had simple suspected intestinal BD, 94 (64.4%) had incomplete type of intestinal BD, and eight (5.5%) had complete type of intestinal BD, based on the established diagnostic criteria. At baseline, 44 cases were treated with colchicine, and 17 cases received 5‐aminosalicylates. We analyzed the clinical outcomes of the entire cohort, including both patients with intestinal BD and those with simple ulcers.

**TABLE 2:** Clinical characteristics of patients with intestinal Behçet's disease and simple ulcer.

Median age (years) (IQR)	47 (35.61)
Sex (female, %)	57 (39.0%)
Disease phenotype	
Simple ulcer (%)	18 (12.3%)
Suspected intestinal Behçet's disease (%)	26 (17.8%)
Incomplete type of intestinal Behçet's disease (%)	94 (64.4%)
Complete type of intestinal Behçet's disease (%)	8 (5.5%)
Severity of intestinal Behçet's disease before treatment	
Remission (%)	2 (1.4)
Mild (%)	9 (6.2)
Moderate (%)	48 (32.9)
Severe (%)	87 (59.6)
Endoscopic findings of ulcerative lesions before treatment	
Remission (%)	3 (2.1)
Mild (%)	25 (17.1)
Moderate (%)	42 (28.8)
Severe (%)	76 (52.1)
Disease activity index for intestinal Behçet's disease (DAIBD) before treatment	
≤ 19: Quiescent (%)	16 (11.0)
20–39: Mild (%)	17 (11.6)
40–74: Moderate (%)	41 (28.1)
≥ 75: Severe (%)	72 (49.3)
Median DAIBD before treatment	70 (40.105)
Concomitant medications	
5‐aminosalytilate (%)	17 (11.6)
Colchitine (%)	44 (30.1)
Thiopurine (%)	9 (6.2)
Methotrexate (%)	3 (2.1)

Abbreviation: IQR, interquartile range.

The proportion of cases with severe disease among the patients with each type of BD was comparable. However, 25% of patients with complete BD had mild disease, while a few cases with incomplete BD (5.3%), suspected BD (3.8%), and simple ulcer (5.6%) had mild disease (Table S1).

**FIGURE 2:**
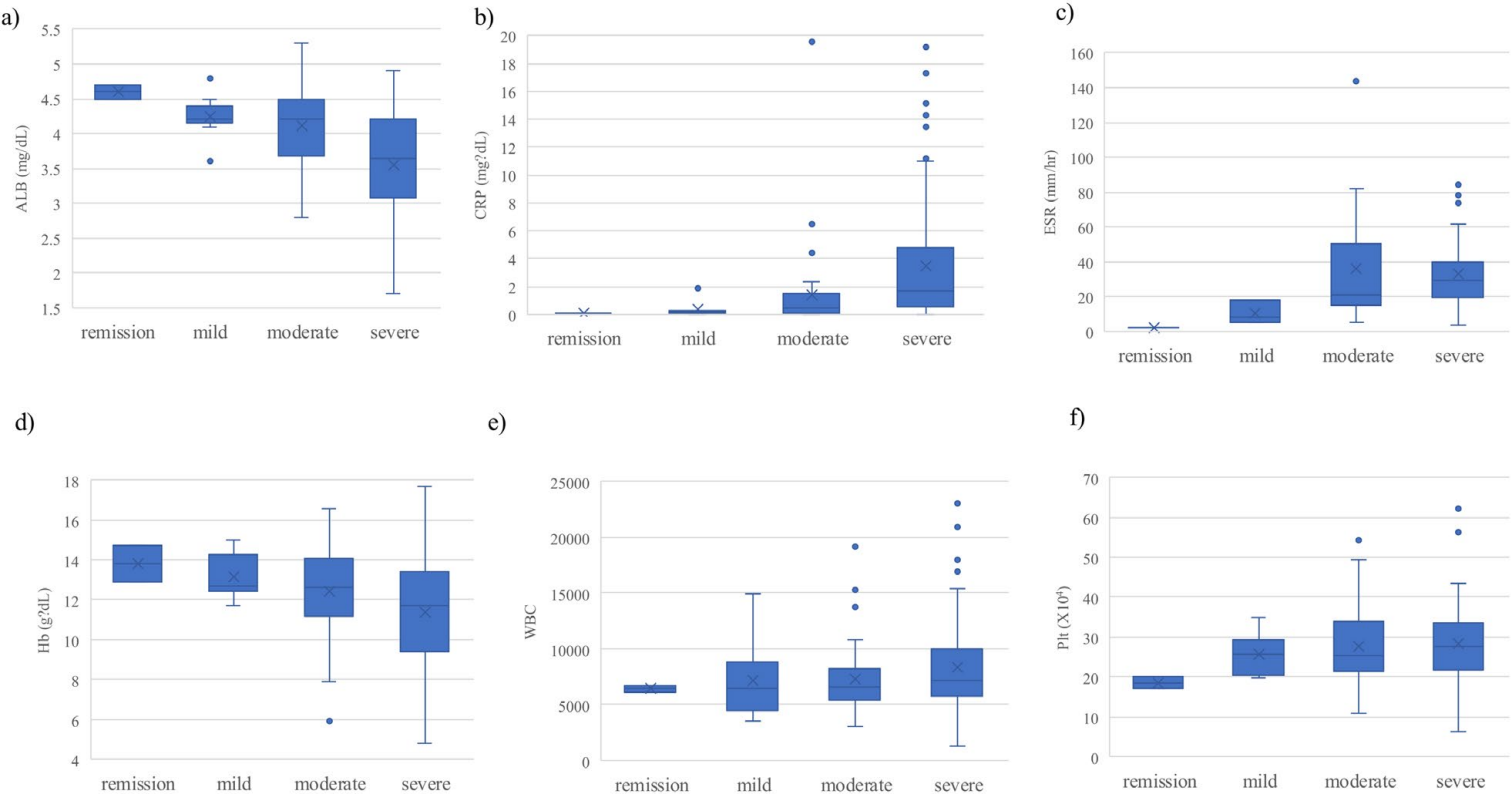
Correlations between severity criteria for intestinal BD (SCIBD) and (a) serum albumin (ALB), (b) C‐reactive protein (CRP), (c) erythrocyte sedimentation rate (ESR), (d) hemoglobin (HB), (e) white blood cell (WBC) count, and (f) platelet count (PLT) were assessed in the overall population, including patients with intestinal Behçet's disease and simple ulcers.

We assessed the relationship between disease severity and blood biomarker levels in the study population before treatment (Table 3). As shown in Figure 2a and Table 3, the median ALB level was significantly different among patients with remission, mild, moderate, and severe disease in SCIBD (*p* < 0.001). CRP (Figure 2b and Table 3 *p* < 0.001), ESR (Figure 2c and Table 3, *p* = 0.043), and HB levels (Figure 2d and Table 3, *p* = 0.036) were also significantly different among the groups. However, the median WBC (*p* = 0.394) and PLT (*p* = 0.296) counts were not significantly different between the groups (Figure 2e,f and Table 3).

**TABLE 3:** Comparison of disease severity with biomarkers and Japanese severity criteria for intestinal BD in the whole population (*n* = 146).

	Remission (*n* = 2)	Mild (*n* = 9)	Moderate (*n* = 48)	Severe (*n* = 87)	*p*
Alb (g/dL) (IQR)	4.6	4.2 (4.15, 4.4)	4.2 (3.675, 4.5)	3.65 (3.1, 4.2)	< 0.001
TP (g/dL) (IQR)	7.7	6.9 (6.6, 7.25)	7.3 (6.9, 7.6)	6.9 (6.1, 7.5)	0.017
CRP (mg/dL) (IQR)	0.04	0.13 (0.05, 0.26)	0.48 (0.12, 1.46)	1.71 (0.60, 4.79)	< 0.001
ESR (mm/h) (IQR)	2	8 (5, –)	21 (15, 50.5)	29 (19, 40)	0.043
WBC count (/μL)	6445	6400 (4400, 8800)	6615 (5450, 8172)	7200 (5800, 10,400)	0.394
Hb (g/dL) (IQR)	13.8	12.7 (12.5, 14.3)	12.6 (11.2, 14.1)	11.7 (9.4, 13.4)	0.036
Platelet count (×10^4^/μL)	18.6	25.5 (10.3, 29.4)	25.4 (21.3, 34.0)	27.5 (21.6, 33.5)	0.296
DAIBD	15	20 (5, 62.5)	50 (20, 85)	90 (55, 120)	< 0.001

Abbreviations: Alb, albumin; CRP, C‐reactive protein; DAIBD, disease activity for intestinal Behçet's disease; ESR, erythrocyte sedimentation rate; Hb, hemoglobin; IQR, interquartile range; TP, total protein; WBC, white blood cell.

For the sensitivity analysis, only patients with intestinal BD (excluding those with simple ulcers; *n* = 102) were included. Similar trends were observed for the relationship between disease severity and biomarkers in this intestinal BD population. As the disease severity increased, CRP levels increased significantly, whereas ALB levels decreased. In contrast, no significant associations were found between disease severity and other markers (Figure S1 and Table S2).

### 3.2 Comparison of DAIBD With SCIBD Before Treatment

**FIGURE 3:**
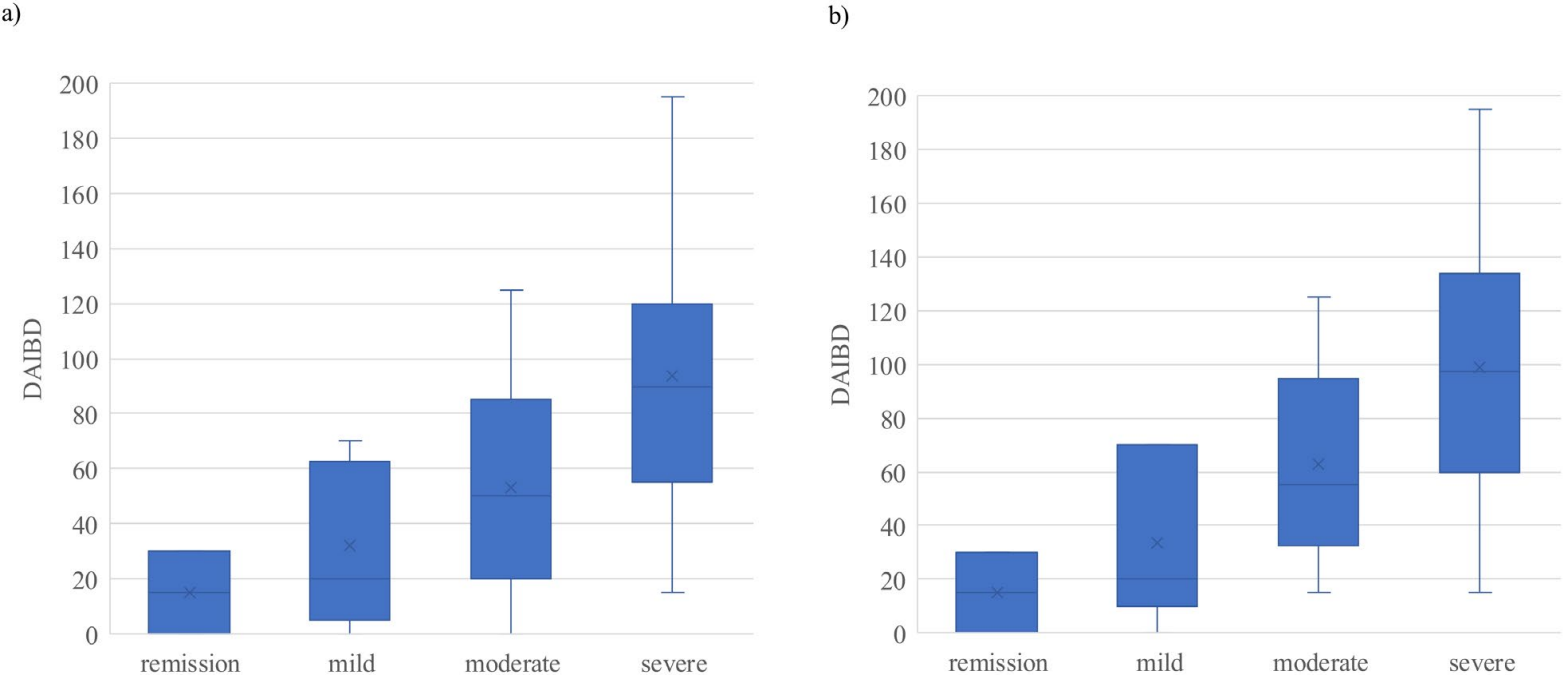
Correlations between severity criteria for intestinal BD (SCIBD) and disease activity for intestinal BD (DAIBD) in the whole population (a) and in patients with intestinal Behçet's disease alone (b).

We assessed the relationship between the DAIBD scores and SCIBD. The DAIBD scores tended to increase as the severity of SCIBD increased (*p* < 0.001)in the entire population (Figure 3a and Table 3). A similar trend was observed even in patients with intestinal BD (*p* < 0.001) (Figure 3b).

Subsequently, we examined the concordance between the SCIBD and DAIBD in terms of disease severity. Disease severity was consistent in 74 cases (50.7%) in our cohort (Table S3). Approximately two‐thirds of the patients (65.5%, 57/87) with severe disease according to the SCIBD, were classified as having severe disease according to the DAIBD classification. Among these patients, 7 out of 87 patients with severe disease (8.0%) and 19 out of 48 patients with moderate disease (39.6%), as classified by SCIBD, were classified as being in remission or having mild DAIBD. Conversely, out of 10 remitting cases that were judged by DAIBD, 9 were judged to have moderate disease based on endoscopic findings on SCIBD and the other two remitting cases of DAIBD had deep ulcers that were deemed severe, according to SCIBD.

### 3.3 Relationship Between Severity Assessment and Treatment Decision/Prognosis

**FIGURE 4:**
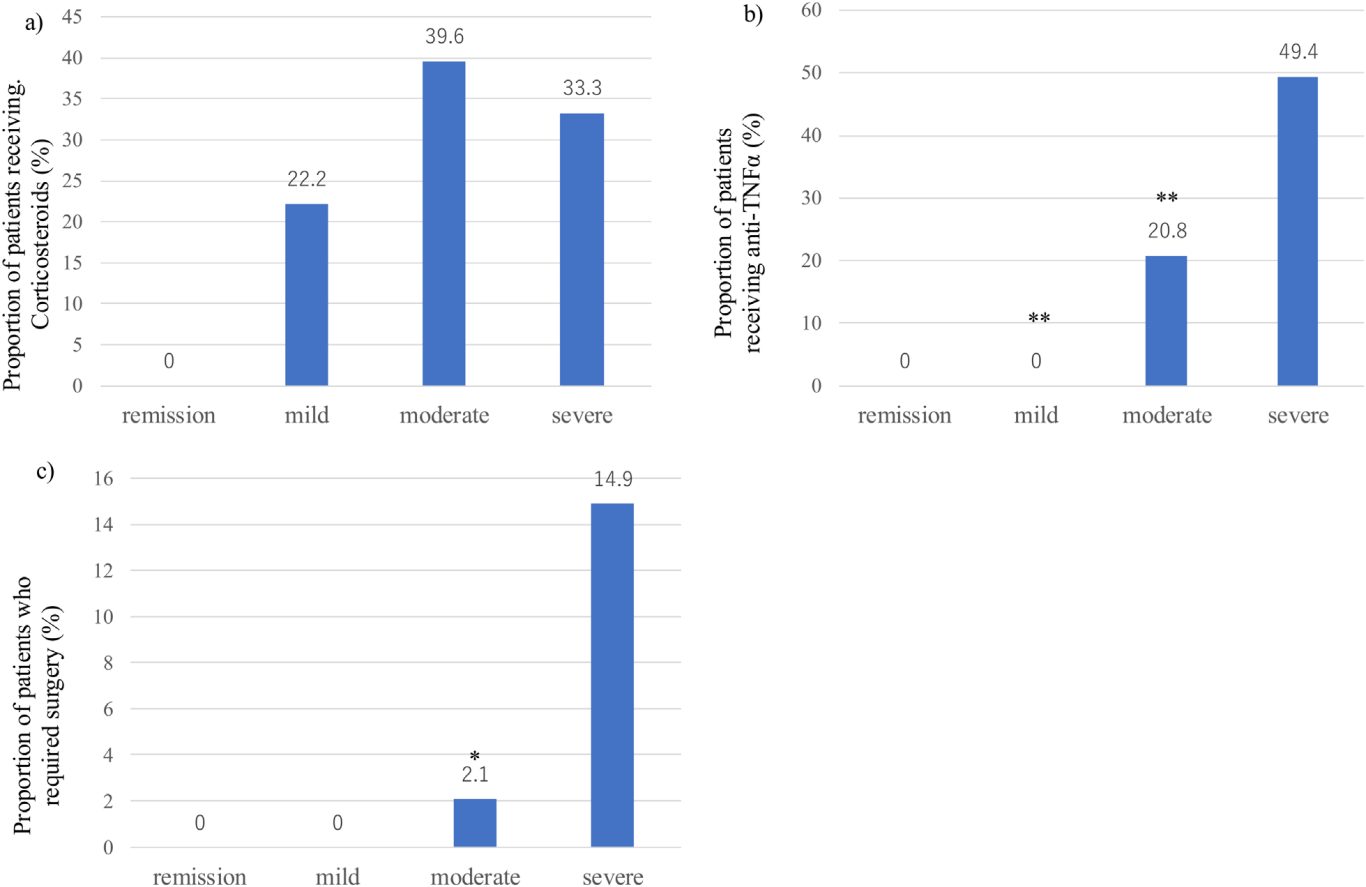
Proportion of patients who received (a) corticosteroids or (b) antitumor necrosis factor‐α, (c) patients who needed surgery in the whole population. *Asterisks* denote a significant difference between each severity (remission, mild, moderate disease) and the severe disease (Fisher's exact test, *p < 0.05; **p < 0.01; ***p < 0.001).

In cases of remission or mild SCIBD, no patients required anti‐TNF‐α treatment or surgery (Table 4 and Figure 4). Among those with mild cases, two out of nine (22.2%) received corticosteroid treatment. Anti‐TNF‐α treatment was also significantly more common in severe cases (49.4%) than in mild (0.0%; *p* = 0.003) or moderate (20.8%; *p* = 0.001) cases (Table 4 and Figure 4). The proportion of patients who received corticosteroids was comparable between the moderate (39.6%) and severe disease groups (33.3%) (*p* = 0.468).

**TABLE 4:** Treatment selection and prognosis in patients with remission, mild, moderate, and severe disease classified by disease severity.

	Disease severity			
	Remission (*n* = 2)	Mild (*n* = 9)	Moderate (*n* = 48)	Severe (*n* = 87)
Use of corticosteroids	0 (0.0%)	2 (22.2%)	19 (39.6%)	29 (33.3%)
*p* value (for severe disease)	0.452	0.497	0.468	
*p* value (for moderate disease)	0.380	0.276		
Use of anti‐TNF‐α treatment	0 (0.0%)	0 (0.0%)	10 (20.8%)	43 (49.4%)
*p* value (for severe disease)	0.264	0.003	0.001	
*p* value (for moderate disease)	0.637	0.132		
Necessity of surgery	0 (0.0%)	0 (0.0%)	1 (2.1%)	13 (14.9%)
*p* value (for severe disease)	0.728	0.212	0.014	
*p* value (for moderate disease)	0.960	0.842		

Abbreviation: anti‐TNF‐α, antitumor necrosis factor‐alpha.

For surgery, the median duration from assessment of the severity of intestinal BD to surgery was 29.5 days (IQR: 5.0–58.25). The indications for surgery included perforation (*n* = 7), massive gastrointestinal bleeding (*n* = 4), refractoriness to medical treatment (*n* = 2), and intestinal stenosis (*n* = 1). The proportion of patients requiring surgery was significantly higher in those with severe disease (14.9%) than in those with moderate disease (2.1%) (*p* = 0.014) (Table 4 and Figure 4c).

Next, we assessed the relationship between DAIBD scores, treatment decisions, and prognosis (Figure S2 and Table 5). The proportion of patients who required corticosteroids, anti‐TNF‐α treatment, and surgery was higher in severe cases (Table 5). However, no significant differences were observed in the frequency of corticosteroid treatment (Figure S2a), anti‐TNF‐α treatment (Figure S2b), or surgery (Figure S2c) among the four groups: quiescent, mild, moderate, and severe cases.

**TABLE 5:** Treatment selection and prognosis in patients with remission, mild, moderate, and severe disease classified by DAIBD.

	Disease severity			
	Remission (*n* = 16)	Mild (*n* = 17)	Moderate (*n* = 41)	Severe (*n* = 72)
Use of corticosteroids	3 (18.8%)	6 (35.3%)	13 (31.7%)	28 (38.9%)
*p* value (for severe disease)	0.106	0.784	0.445	
*p* value (for moderate disease)	0.263	0.791		
Use of anti‐TNF‐α treatment	3 (18.8%)	5 (29.4%)	14 (34.1%)	31 (43.1%)
*p* value (for severe disease)	0.061	0.227	0.352	
*p* value (for moderate disease)	0.381	0.489		
Necessity of surgery	0 (0.0%)	0 (0.0%)	3 (7.3%)	11 (15.3%)
*p* value (for severe disease)	0.094	0.083	0.175	
*p* value (for moderate disease)	—	0.364		

Abbreviations: anti‐TNF‐α, anti‐tumor necrosis factor‐alpha; DAIBD, disease activity for intestinal Behçet's disease.

### 3.4 Changes in Disease Classification Before and After Treatment With Corticosteroids and TNF‐α

**FIGURE 5:**
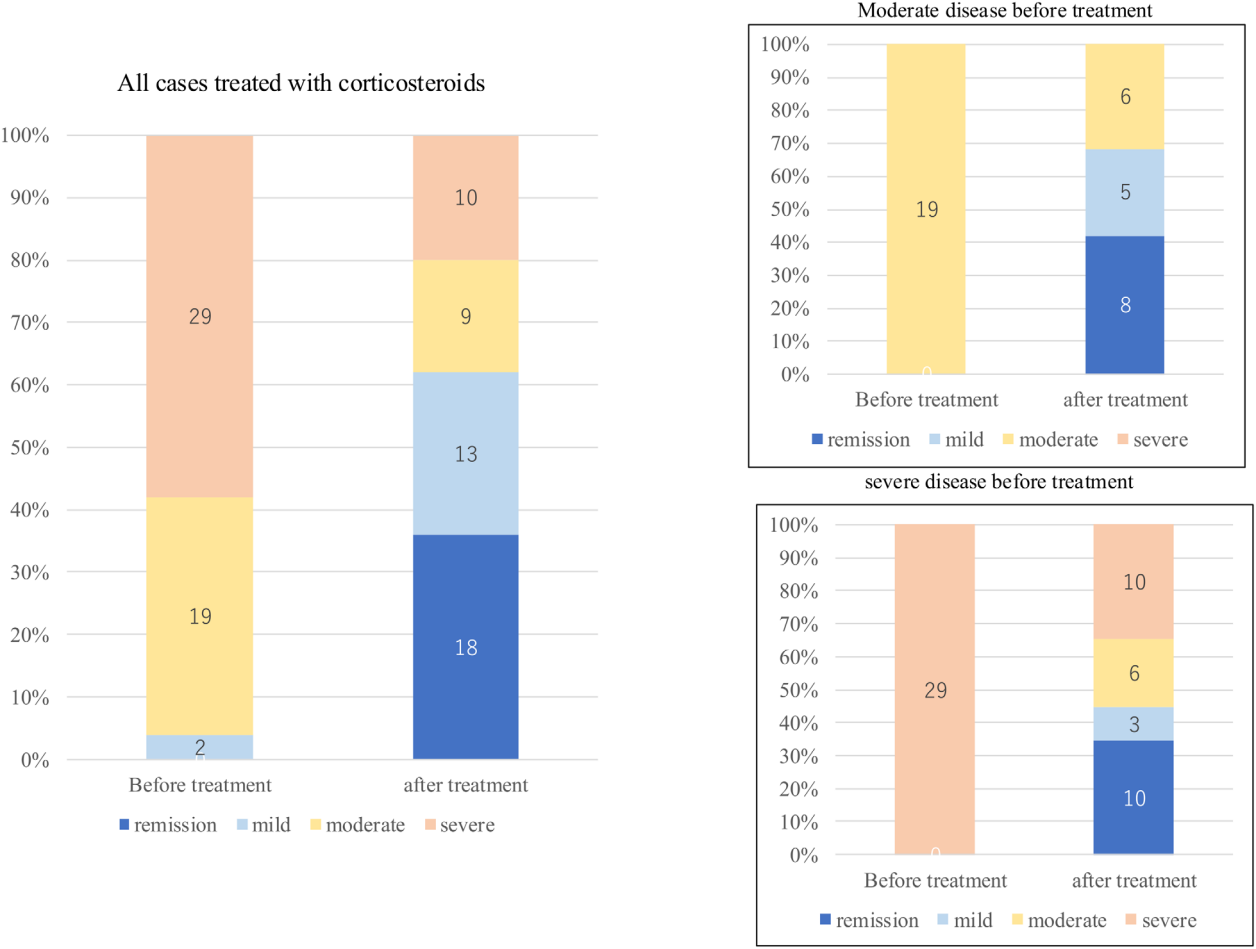
Changes in severity classification before and after corticosteroid treatment and cases with moderate or severe disease based on SCIBD before treatments.

**FIGURE 6:**
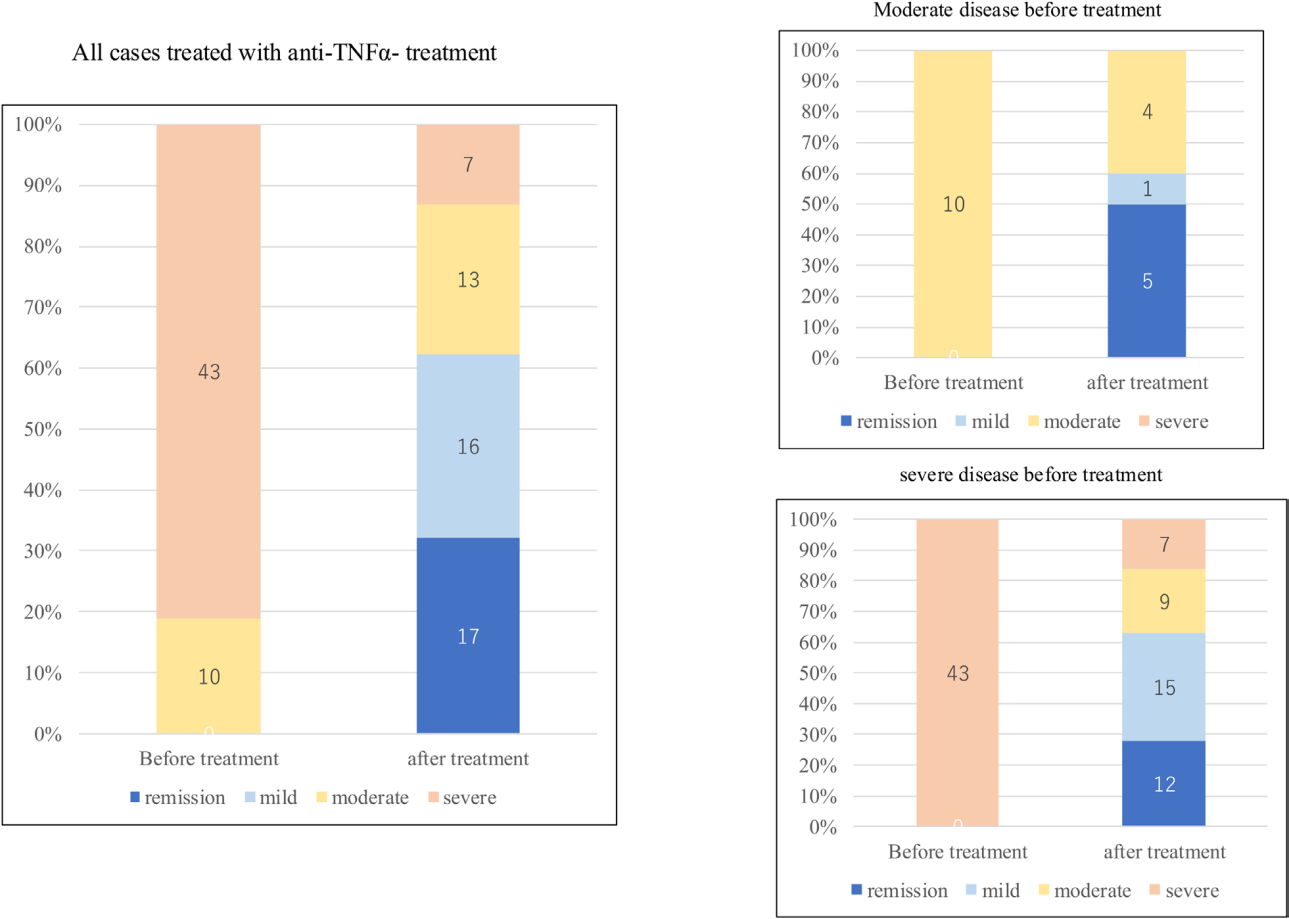
Changes of severe classification before and after anti‐TNF‐α treatment in all cases and cases with moderate or severe disease based on SCIBD before treatments.

Among the patients who received corticosteroids following the initial colonoscopy, 8 (42.1%) and 5 (26.3%) of the 19 patients with moderate disease demonstrated improvement in remission and mild disease, respectively (Figure 5). Among patients treated with anti‐TNF‐α agents after the first colonoscopy, 12 (27.9%) and 15 (34.9%) of the 43 patients with severe disease showed improvement in remission and mild disease, respectively (Figure 6). Furthermore, 5 (50.0%) of the 10 patients with moderate disease improved to remission, whereas 1 (10.0%) improved to mild disease. Notably, 13 (44.8%) of the 29 patients with severe disease achieved remission or mild disease after receiving corticosteroids, whereas 27 (62.8%) of the 43 patients with severe disease achieved remission or mild disease after receiving anti‐TNF‐α agents.

## 4 Discussion

In this study, we established a new disease severity measure for intestinal BD, SCIBD, which consists of five items: clinical symptoms, CRP levels, and endoscopic severity. The SCIBD score was closely correlated with other inflammatory markers, serum albumin levels, and the DAIBD score, the former severity score for intestinal BD. Additionally, our findings indicate that the SCIBD score correlates with appropriate treatment decisions and prognosis prediction. Importantly, SCIBD is composed of items that include endoscopic severity, and it is notable in that it can be determined to be severe based solely on the presence of deep ulceration. Because intestinal BD can lead to intestinal perforation, it is important to determine disease severity, including endoscopic findings, even in asymptomatic patients. SCIBD may be useful for assessing the severity of patients with intestinal BD in a clinical setting.

Severe manifestations of BD are often associated with multiple relapses and poor prognosis, particularly in patients requiring surgical intervention [8, 9]. Therefore, accurate assessment of intestinal BD severity is critical in the clinical settings. Although several tools exist to evaluate disease severity in ulcerative colitis and Crohn's disease, the DAIBD remains the only validated tool for assessing the severity of intestinal BD. Previous studies have indicated that the DAIBD score is associated with both endoscopic severity and prognosis [10, 11, 12]. Specifically, the total number of ulcers and volcano‐shaped ulcers were associated with higher DAIBD scores [10]. In terms of prognosis, a higher DAIBD score at diagnosis is independently associated with a more severe clinical course [11]. Higher CRP levels and DAIBD scores are positively associated with emergency room visits [12].

Although DAIBD is useful in predicting disease prognosis, it does not include endoscopic severity among its items. Predictive factors for poor outcomes include volcano‐shaped deep ulcers, corticosteroid use, and postoperative complications [13]. However, a previous study found that more than half of patients in clinical remission based on DAIBD still had active intestinal inflammation [14]. This suggests that a combination of subjective symptoms, biomarkers, and endoscopic findings may be crucial for assessing the disease severity. Recently, a multidisciplinary group of experts in BD has developed a core set of outcome measures for BD [15]. They recommended that inflammatory markers, endoscopic activity, and clinical gastrointestinal activity should be assessed in clinical trials involving BD. In addition, no definitive criteria exist for assessing the severity of intestinal BD in Japan. Therefore, the research team from the Ministry of Health and Labor's BD Project has been working to establish a novel disease severity index that includes endoscopic findings for intestinal BD. The expert members of this study selected five key subjective symptoms and objective findings associated with the disease severity and clinical outcomes. After establishing the new disease severity, the relationship between SCIBD, biomarkers, and treatment decisions was investigated. Similar to the findings of a previous study on the association between DAIBD and clinical outcomes, our findings indicate that patients classified as having severe cases of SCIBD are more likely to require surgery. Although corticosteroids were mainly used in patients with moderate‐to‐severe SCIBD, the proportion of patients receiving anti‐TNF‐α treatment was significantly higher in patients with severe disease than in those with moderate disease, as defined by SCIBD. Furthermore, no patient in remission or with mild disease received anti‐TNF‐α treatment in our study. These findings are in accordance with clinical guidelines recommending steroids or TNF‐α therapy for cases of moderate or severe disease [4]. In addition, many physicians in our population seem to prefer corticosteroids for moderate cases and steroids or anti‐TNF‐α treatment for severe cases.

CRP levels are elevated due to intestinal inflammation, infection, and inflammation other than intestinal lesions. Determining severe disease based on the elevation of CRP alone may not reflect the activity of intestinal lesions. In this severity classification, the elevation of CRP alone was not considered a severe disease.

SCIBD is considered more useful than DAIBD in guiding the treatment of moderate‐to‐severe disease. Given the recent “treat to target” approach [16, 17], severity assessment, including CRP levels and endoscopy, is necessary, and SCIBD aligns with this concept.

Our study suggests that SCIBD reflects treatment response to corticosteroids and anti‐TNF‐α agents. Among patients with severe disease based on SCIBD, the proportion of patients who achieved remission or mild disease after treatment tended to be higher in the anti‐TNF group than in the steroid group (Figures 5 and 6). This suggests that severity assessment based on the SCIBD may be useful for selecting treatment.

This study had certain limitations. First, the SCIBD was created based on expert consensus and lacked a formal scoring system. However, given the urgent need to determine the severity of the disease, as described in the therapeutic guidelines, we created the SCIBD. Second, the number of patients included in this study was relatively small. However, considering the rarity of intestinal BD compared with Crohn's disease, the inclusion of approximately 150 cases is still significant. Third, the majority of cases in our study were classified as moderate or severe, which may have limited the evaluation of remission and mild symptoms. SCIBD, created based on expert consensus, may prove to be useful for guiding treatment decisions and evaluating the efficacy of medical treatment for intestinal BD in the future. Medical subsidies are available for BD treatment, where subsidies are only available for patients with moderate‐to‐severe disease. Therefore, it was important to determine the disease severity in this study. Future studies including those with patients in remission are necessary to validate these findings further.

In conclusion, the severity assessment of intestinal BD using our novel criteria was correlated with appropriate treatment decisions and prognosis prediction. The integration of additional emerging biomarkers could further enhance the utility of the tool, supporting more personalized and effective management strategies for intestinal BD.

## Author Contributions

M.T. conceived the study. M.N. designed the main concept of this study. M.N., T.F., Y.M., S.B., T.F., M.U., M.M., K.M., K.W., and T.M. designed the criteria for severity of intestinal Behcet Disease. T.F. and M.N. drafted the manuscript and participated in the statistical analysis. T.F., Y.K., R.K., N.U., J.U., S.B., Y.M., M.O., S.H., T.M., C.W., M.N., and M.M. participated in patient enrollment and the acquisition of clinical data. Y.T. and T.H. supervised this project. All the authors contributed to the critical review and approval of the final draft.

## Ethics Statement

The study design was reviewed and approved by the Ethics Committees of Kansai Medical University Hospital (2022066) and each participating institution. An opt‐out approach was used in this study, as the participants were not at risk. Patients were given the opportunity to refuse participation by indicating their preferences on an institutional website. This study was registered with the University Hospital Medical Information Network Center (UMIN000049488; http://www.umin.ac.jp/ctr/). All authors had access to the study data and reviewed and approved the final manuscript.

## Conflicts of Interest

There is no COI directly related to this project. In accordance with DEN submission guidelines, however, COIs for each author that are not directly related to this study are disclosed as below. Makoto Naganuma received grants from AbbVie GK and lecture fees from AbbVie GK and Mitsubishi‐Tanabe Pharmaceutical Co. Ltd. Yohei Kirino received grants from Amagen and lecture fees from Sobi and support for attending meetings from Sobi. Junji Umeno received lecture fees from AbbVie GK, Janssen Pharmaceutical K.K., and Mitsubishi‐Tanabe Pharmaceutical Co. Ltd. Shigeki Bamba received lecture fees from AbbVie GK. Makoto Ooi received lecture fees from AbbVie GK, Kyorin Pharmaceutical Co. Ltd., and Mitsubishi‐Tanabe Pharmaceutical Co. Ltd. Shuhei Hosomi received a lecture fee from Takeda Pharmaceutical Co and AbbVie GK. Takayuki Matsumoto received lecture fees from AbbVie GK, Janssen Pharmaceutical K.K., Takeda Pharmaceutical Co, and Gilead Sciences Inc. and is the responsible and executive JGES member for DEN Open. Katsuyoshi Matsuoka received lecture fees from AbbVie GK and Mitsubishi‐Tanabe Pharmaceutical Co. Ltd. Masakazu Nagahori received lecture fees from AbbVie GK and Mitsubishi‐Tanabe Pharmaceutical Co. Ltd. Kenji Watanabe received lecture fees from AbbVie GK, Takeda Pharmaceutical Co. Ltd., EA Pharma Co. Ltd., Mochida Pharmaceutical Co. Ltd., JIMRO Co. Ltd., and Mitsubishi‐Tanabe Pharmaceutical Co. Ltd., and participated in the advisory Board in Takeda Pharmaceutical Co. Ltd. Fumito Hirai received grants from AbbVie GK and Janssen Pharmaceutical K.K. and lecture fees from AbbVie GK, Janssen Pharmaceutical K.K., and EA Pharma Co. Ltd. Minoru Matsuura received lecture fees from AbbVie GK and Janssen Pharmaceutical K.K. Yoshiya Tanaka received lecture fees from Chugai, UCB, AbbVie, AstraZeneca, Eli Lilly, Behringer‐Ingelheim, GlaxoSmithKline, Eisai, IQVIA, Daiichi‐Sankyo, Otsuka, Taisho, Gilead, and Bristol‐Myers. Tadakazu Hisamatsu received research grants from Mitsubishi Tanabe Pharma Corporation, EA Pharma Co. Ltd., AbbVie GK, JIMRO Co. Ltd., Takeda Pharmaceutical Co. Ltd., Pfizer Inc., and Mochida Pharmaceutical Co. Ltd., and consultant fees from Mitsubishi Tanabe Pharma Corporation, EA Pharma Co. Ltd., AbbVie GK, JIMRO Co. Ltd., Takeda Pharmaceutical Co. Ltd., Pfizer Inc., Bristol Myers Squibb, Abivax, Eli Lilly and Co., and Janssen Pharmaceutical K.K., and received lecture fees from Mitsubishi Tanabe Pharma Corporation, AbbVie GK, Celgene K.K., EA Pharma Co. Ltd., Kyorin Pharmaceutical Co. Ltd., JIMRO Co., Janssen Pharmaceutical K.K., Mochida Pharmaceutical Co. Ltd., Takeda Pharmaceutical Co., Kissei Pharmaceutical Co. Ltd., Nichi‐Iko Co. Ltd., Gilead Sciences Inc., and Pfizer Inc. Other authors declare no conflicts of interest.

## Supporting information


**Table S1:** Distribution of severity category in cases with complete Behçet's disease, incomplete Behçet's disease, suspected Behçet's disease, and simple ulcer.
**Table S2:** Comparison of disease severity with biomarkers and disease activity for intestinal Behçet's disease (DAIBD) in patients with intestinal Behçet's disease (*n* = 102).
**Table S3:** Concordance between disease severity and DAIBD.
**Figure S1:** Correlations between severity criteria for intestinal BD (SCIBD) and (a) serum albumin (ALB), (b) C‐reactive protein (CRP), (c) erythrocyte sedimentation rate (ESR), (d) hemoglobin (HB), (e) white blood cell (WBC) count, and (f) platelet count (PLT) were assessed in patients with intestinal Behçet's disease.
**Figure S2:** Proportion of patients who received (a) corticosteroids or (b) anti‐tumor necrosis factor‐α, (c) patients who needed surgery in patients with intestinal Behçet's alone. *Asterisks* denote a significant difference between each severity (remission, mild, moderate disease) and the severe disease (Fisher's exact test, *p < 0.05; **p < 0.01; ***p < 0.001).

## Data Availability

The datasets generated and analyzed during the study are not publicly available due to a lack of permission for external access. However, the data can be obtained from the corresponding author upon reasonable request, subject to permission from the Ethics Committee of Kansai Medical University.
