# Supplementary material for: A Multicenter Observational Study for the Establishment of Novel Severity Criteria Including Endoscopic Evaluation for Intestinal Behçet's Disease
Source: Dig Endosc. 2025 Sep 27;38(1):e70041. doi: 10.1111/den.70041 (PMC12746405; doi:10.1111/den.70041)
Supplement: Supplementary file 1 — Table S1: Distribution of severity category in cases with complete Behçet's disease, incomplete Behçet's disease, suspected Behçet's disease, and simple ulcer. Table S2: Comparison of disease severity with biomarkers and disease activity for intestinal Behçet's disease (DAIBD) in patients with intestinal Behçet's disease (n = 102). Table S3: Concordance between disease severity and DAIBD. Figure S1: Correlations between severity criteria for intestinal BD (SCIBD) and (a) serum albumin (ALB), (b) C‐reactive protein (CRP), (c) erythrocyte sedimentation rate (ESR), (d) hemoglobin (HB), (e) white blood cell (WBC) count, and (f) platelet count (PLT) were assessed in patients with intestinal Behçet's disease. Figure S2: Proportion of patients who received (a) corticosteroids or (b) anti‐tumor necrosis factor‐α, (c) patients who needed surgery in patients with intestinal Behçet's alone. Asterisks denote a significant difference between each severity (remission, mild, moderate disease) and the severe disease (Fisher's exact test, *p < 0.05; **p < 0.01; ***p < 0.001). [file DEN-38-0-s001.docx]

**Supplemental Information**

**Establishment of Severity Criteria for Intestinal BD**

Severity criteria for intestinal BD was established First, the primary investigators at Kansai Medical Dental University (FT and MN) extracted candidate items to assess the clinical severity of Intestinal BD. We considered it appropriate for the evaluation of severity to combine subjective and objective items and endoscopic activity. Accordingly, the following items were extracted with reference to the DAIBD items: abdominal pain, abdominal tenderness, number of diarrhea, intestinal bleeding, fever, intestinal complications (fistula, abscess, and perforation), serum CRP, hemoglobin, and endoscopic severity. An expert team ((MN, FT, YM, SB, FH, MU, MM, KM, KW, and TM) discussed how to narrow down the candidate items for severity assessment. After discussion, diarrhea cases were excluded as they were considered unlikely to occur with lesions limited to the ileocecal ulceration, which is commonly observed in patients with intestinal BD. The item for hemoglobin levels may not necessarily reflect the activity of gastrointestinal lesions (e.g., menstruation), and assessing gastrointestinal bleeding would better reflect the activity. Items for fever were excluded because they were not specific to intestinal BD. Finally, abdominal pain and intestinal bleeding were selected as subjective symptoms, whereas abdominal tenderness, serum CRP levels, and endoscopic findings were chosen as objective indicators (Table 1). On the basis of expert meetings, each item was categorized into three grades: 0, 1, and 2. Abdominal pain was classified as follows: “no pain” (grade 0), “mild pain that does not interfere with daily life” (grade 1), and “moderate pain that interferes with daily life” (grade 2). For abdominal tenderness, grade 0 indicated no tenderness, whereas grade 2 indicated tenderness without peritoneal signs. Intestinal bleeding was categorized as either “no bleeding” (grade 0) or “overt bleeding from the anus” (grade 2). Serum CRP levels were classified according to the following criteria: grade 0 for a normal CRP level, grade 1 for a CRP level above the standard threshold but less than 1.0 mg/dL, and grade 2 for CRP levels ≥1.0 mg/dL. Regarding endoscopic findings, aphthae and ulcers less than 1 cm in size were defined as grade 1, whereas well-demarcated, shallow ulcers ≥1 cm were defined as grade 2 (Figure 1).

Patients in remission were defined as those who met all five item conditions at grade 0. Mild disease was classified as the presence of at least one item in grade 1, without any items in grade 2 or higher. Moderate disease was defined as the presence of at least one item at grade 2, excluding any severe items. Severe disease was defined as the presence of at least one item of the grade 3 following clinical symptoms, objective findings, or imaging findings: severe pain that interferes with daily life; abdominal tenderness with peritoneal signs; massive bleeding accompanied by lower blood pressure or requiring blood transfusion; deep ulceration with a sharply demarcated (Figure 1), cliff-cut margin; intra-abdominal abscess; or intestinal penetration or perforation. In this definition, blood transfusion refers to the transfusion of red blood cells due to anemia.

After drafting the preliminary severity criteria, feedback from the members of the Health and Labor Sciences Research Grants for Research on Intractable Diseases was incorporated. The final version was created and finalized through consensus.

**Supplemental Table 1:** Distribution of severity category in cases with complete Behçet’s disease (BD), incomplete BD, suspected BD, and simple ulcer.

|  | Severity category | | | |
| --- | --- | --- | --- | --- |
|  | Remission | Mild | Moderate | Severe |
| Complete BD | 0 (0.0%) | 2 (25.0%) | 2 (25.0%) | 4 (50.0%) |
| Incomplete BD | 2 (2.1%) | 5 (5.3%) | 27 (28.7%) | 60 (63.8%) |
| Suspected BD | 0 (0.0%) | 1 (3.8%) | 13 (50.0%) | 12 (46.2%) |
| Simple ulcer | 0 (0.0%) | 1 (5.6%) | 6 (33.3%) | 11 (61.1%) |

**Supplemental Table 2:** Comparison of Japanese severity for intestinal Behçet’s disease with biomarkers and disease activity for intestinal Behçet’s disease (DAIBD) in patients with intestinal Behçet’s disease (n=102)

|  | Japanese severity for intestinal Behçet’s disease (JSIBD) | | | |  |
| --- | --- | --- | --- | --- | --- |
|  | Remission  (n=2) | Mild  (n=7) | Moderate  (n=29) | Severe  (n=64) | P value |
| Alb (mg/dL) (IQR) | 4.6 | 4.2 (4.1, 4.3) | 4.1 (3.6, 4.5) | 3.7 (3.2, 4.2) | 0.002 |
| TP  (mg/dL) (IQR) | 7.7 | 6.9 (6.6, 7.4) | 7.4 (6.9, 7.7) | 6.8 (6.25, 7.425) | 0.029 |
| CRP  (mg/dL) (IQR) | 0.04 | 0.13 (0.02, 0.30) | 0.73 (0.13, 2.07) | 1.82 (0.60, 5.07) | <0.001 |
| ESR  (mm/hr) (IQR) | 2 | 13 (8, -) | 28.5 (16.75, 66.25) | 28.5 (19.25, 40) | 0.128 |
| WBC count  (/μL) | 6445 | 6,510 (5,100, 9,800) | 7,110 (5,550, 9,445) | 8,100 (6,150,10,500) | 0.419 |
| Hb  (g/dL) (IQR) | 13.8 | 12.7 (12.4, 14.4) | 12.2 (11.1, 13.5) | 12.1 (9.7,13.4) | 0.198 |
| Platelet count (×10^4^/μL) | 18.6 | 28.1 (19.9, 29.8) | 26.8 (20.8, 36.5) | 29.6 (22.7, 33.7) | 0.254 |
| DAIBD | 15 | 20 (10, 70) | 55 (32.5, 95) | 97.5 (60, 133.75) | <0.001 |

Alb, albumin; TP, total protein; CRP, C-reactive protein; WBC, white blood cell; Hb; Hemoglobin, DAIBD, disease activity for intestinal Behçet’s disease; ESR, erythrocyte sedimentation rate; IQR, interquartile range

**Supplemental Table 3:** The concordance between the severity of intestinal Behçet’s disease and DAIBD

|  | | Severity for intestinal Behçet’s disease | | | |
| --- | --- | --- | --- | --- | --- |
|  |  | Remission | Mild | Moderate | Severe |
| DAIBD | Remission | 1 | 3 | 10 | 2 |
|  | Mild | 1 | 2 | 9 | 5 |
|  | Moderate | 0 | 4 | 14 | 23 |
|  | Severe | 0 | 0 | 15 | 57 |

DAIBD, disease activity for intestinal Behçet’s disease

**Supplemental Figure 1:** Correlations between Japanese severity criteria for intestinal BD (JSIBD) and a) serum albumin (ALB), b) C reactive protein (CRP), c) erythrocyte sedimentation rate (ESR), d) hemoglobin (HB), e) white blood cell (WBC) count and f) platelet count (PLT) were assessed in patients with intestinal Behçet’s disease.

**Supplemental Figure 2:** Proportion of patients who received a) corticosteroids or b) anti-tumor necrosis factor-α, or c) surgery in patients with intestinal Behçet’s alone who had remission, mild, moderate, and severe disease based on DAIBD

*Asterisks* denote a significant difference between each severity (remission, mild, moderate disease) and the severe disease (Fisher’s exact test, ^∗^ *p*<0.05; ^∗∗^ *p*<0.01; ^∗∗∗^ *p*<0.001)
